# A feasibility study of multislice X‐ray CT imaging of gel dosimeters using the “zero scan” method

**DOI:** 10.1120/jacmp.v15i4.4360

**Published:** 2014-07-08

**Authors:** Muhammad B. Kakakhel, Tanya Kairn, Paul Charles, Jamie V. Trapp

**Affiliations:** ^1^ Department of Physics and Applied Mathematics DPAM, Pakistan Institute of Engineering and Applied Sciences Islamabad Pakistan; ^2^ Premion Cancer Care The Wesley Medical Centre Auchenflower Australia; ^3^ School of Chemistry Physics & Mechanical Engineering, Queensland University of Technology Brisbane Australia

**Keywords:** volumetric reconstruction, gel dosimeters, radiation therapy, X‐ray computed tomography

## Abstract

This study extends the ‘zero scan’ method for CT imaging of polymer gel dosimeters to include multislice acquisitions. Multislice CT images consisting of 24 slices of 1.2 mm thickness were acquired of an irradiated polymer gel dosimeter and processed with the zero scan technique. The results demonstrate that zero scan‐based gel readout can be successfully applied to generate a three‐dimensional image of the irradiated gel field. Compared to the raw CT images, the processed figures and cross‐gel profiles demonstrated reduced noise and clear visibility of the penumbral region. Moreover, these improved results further highlight the suitability of this method in volumetric reconstruction with reduced CT data acquisition per slice. This work shows that 3D volumes of irradiated polymer gel dosimeters can be acquired and processed with X‐ray CT.

PACS number: 87.57.Q‐, 87.57.nf, 87.55.‐x

## I. INTRODUCTION

Radiotherapy dose verification is an integral part of the cancer treatment chain. Polymer gel dosimeters offering 3D dose verification capabilities have been developed^(1)^ for the verification of complex radiotherapy treatments.

Readout of dose information from polymer gel dosimeters can be undertaken with imaging techniques, such as magnetic resonance imaging (MRI)^2^, ^3^, ^4^ and optical computed tomography (CT),^5^, ^6^, ^7^ though these modalities are not available at all radiation oncology clinics. X‐ray CT, which is commonly available in radiotherapy departments, is a viable alternative for scanning gel images, despite suffering from poor signal‐to‐noise ratio owing to a relatively small increase in gel density.^8^, ^9^ Recently ‘zero scan’ method, a simple technique for X‐ray CT of polymer gel, has been introduced, offering improved image quality.^(10)^ The ‘zero scan’ method generates a new image as follow: 1) repeat a number of CT scans of the irradiated gel phantom; 2) due to the massive number of CT scans, the absorbed dose will increase, which gives an increased Hounds field number (HU) with increasing number of scans; 3) a function is fitted on a pixel‐by‐pixel basis for the fractional increase in the gel density with each scan; 4) the intercept value of the fit for each pixel forms the new image (i.e., the ‘zero scan’).

In the present study, the zero scan method introduced by Kakakhel et al.^(10)^ and previously demonstrated for a single two‐dimensional CT slice, has been used for the three‐dimensional volumetric reconstruction of a multislice irradiated gel, while reducing the number of CT scans required for high‐contrast imaging.

## II. MATERIALS AND METHODS

A PAGAT gel dosimeter according to the recipe of Venning et al. (who studied PAGAT gel dosimeters using magnetic resonance imaging)^(3)^ was prepared with increased concentration (8 mM) of Tetrakis (hydroxymethyl) phosphonium chloride (THPC) for improved stability, as suggested by Khoei et al. in their investigation of the pre‐irradiation temporal stability of PAGAT gel dosimeter.^(11)^ The gel was poured into a cylindrical polyethylene terephthalate (PET) container and stored at 4°C for 15 hours prior to irradiation. Using an open field of $3\times 3\;{\text{cm}}^{2}$ a total of 1300 cGy was delivered to the gel container on a Varian iX linear accelerator (Varian Medical Systems, Palo Alto, CA), with 6 MV photon beams (at a dose rate of 600 MU/min). A treatment plan to deliver the dose to the phantom was not prepared; however, the gantry angle and isocenter position used in the irradiation were selected to give a suitably wide range of doses to the gel.

Postirradiation storage of the gel was carried out at room temperature. The X‐ray CT of the gel was conducted 2 h after irradiation in two steps. First the gel container was placed in a cylindrical water phantom and a total of 24 contiguous 1.2 mm images were scanned using a Siemens Sensation CT scanner (Siemens AG Medical Solutions, Waukesha, Germany). The X‐ray tube load of 300 mA with a 1 s rotation, a nominal tube energy of 120 kVp, 24×1.2 mm slices, and a 30 cm scan field of view reconstructed on a 512×512 pixel image matrix was used. The gel scan for the entire 24 slices was repeated 72 times, thus producing a total of 24×72 DICOM images. In the second step, the gel container was removed from the phantom and water only images were acquired, keeping the imaging parameters unchanged. The water images were averaged together (simply by adding all the images matrices and dividing by the total number of images) and used further by subtracting the averaged water image from each gel scan to remove CT artifacts. The averaged water images were subtracted from the respective gel slices, as suggested by Trapp et al.^(8)^ for the removal of CT artifacts (Trapp and colleagues have experimentally studied the dose response of polymer gel dosimeters imaged with X‐ray computed tomography).^(8)^ MATLAB (version 7.13.0.564, MathWorks, Natick, MA) was used to process the image to generate zero scan images in a similar fashion in which Kakakhel et al.^(10)^ produced single slices. Individual slices were reconstructed by applying the linear fit, as described earlier, and stacked together for volumetric representation.^(10)^ For linear fitting, generic MATLAB function polyfit was used, whereas for the generation of the isosurface plots, MATLAB's Sliceomatic script was used.

## III. RESULTS

**Figure 1:**
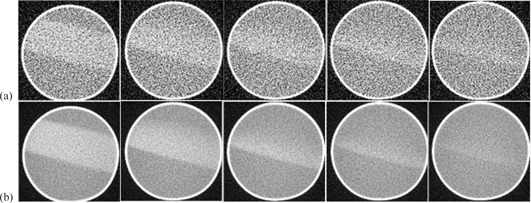
Raw gel images (a) from slice 1, 16‐19, and zero scan gel images (b) from slice 1, 16–19. All images are windowed to the range 0–40 HU.

**Figure 2:**
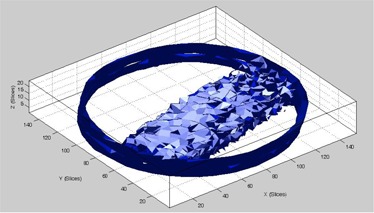
Irradiated gel volume indicated by the isosurface using the zero scan images.

**Figure 3:**
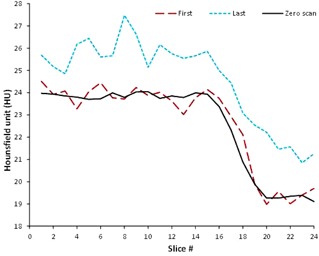
Profiles through the irradiated gel using an ROI of 10×10.

Figure 1 shows the transverse raw and zero scan images from a few selected slices. Raw images correspond to the first gel acquisition of each image set. In Fig. 1, panels A & B show the raw and zero scan slices, respectively, for slice 1, and slices 16‐19 which show the penumbral region. It can be clearly observed that the zero scan images have reduced noise. For quantification of the reduction in noise, an ROI of 26×26 pixels (from the irradiated gel portion) was selected from the raw slice 1 and the corresponding zero scan image, and the standard deviation in the processed image was reduced by half. Slices 16‐19 clearly show the top edge of the field progressively diminishing as the penumbral region is traversed. This information is not clearly visible in the raw image; however, in the corresponding zero scan image in panel B, this can clearly be appreciated. Similarly from slice 16–19 in panel B, the zero scan images are indicating the diminishing penumbra region, where this information is difficult to extract from the respective raw CT images in panel A. Figure 2 shows an isosurface plot drawn in MATLAB where the irradiated volume is clearly identifiable, demonstrating the ability of zero scan method for the volumetric reconstruction of gel images. Figure 3 shows the first, last, and zero scan profiles across a 10×10 region of interest (ROI) over the entire 24 slices. First and last refers to the first (image 1) and last (image 72) gel acquisition, respectively, in any particular slice. It can be clearly seen from the profiles that the zero scan profile has a reduced noise with well‐defined penumbral region. Comparing the all the three profiles for first 14 slices (excluding the penumbral region), the standard deviation was found to be 0.13, 0.06, and 0.27 (CT numbers) for first, zero scan, and last slices, respectively.

## IV. DISCUSSION & CONCLUSION

The results presented in Figs. 1–3 demonstrate the ability of the zero scan method for improved gel dose readout in agreement with previously published work^(10)^ In this study, multislice CT scanning of the gel has been performed and has been shown to work.

One consideration is the scatter arising in a multislice CT scan (i.e., the computed tomography dose index (CTDI)). As in the original study by Kakakhel et al.,^(10)^ a single slice was repeatedly scanned and reconstructed; however, in contrast, here the entire gel was scanned where the scatter will affect the radiation history of the neighboring slices. However, because a zero‐scan (3D) image is generated from the intercept of each pixel's line fit rather than the gradient, the different CT dose received by each pixel during scanning will not cause discrepancies in the final image.

Acrylamide‐based gel has its limitations and uncertainties related to temperature and other factors. However, the purpose of this manuscript is to describe the imaging method as a feasibility study, and a full investigation of all uncertainties associated with CT gel dosimetry are left for future work. In fact, an optimization of CT for gel dosimetry would naturally include a change of gel dosimeter to NIPAM gels, and the scope of such a study would be beyond the scope of this current work. Therefore, it is preferred to keep the current study as a feasibility work on the extension of the imaging method to multislice CT, implying it's potential use in cone‐beam CT.

Regarding the selection of CT parameters, a high mAs was selected so that the CT dose delivered to the gel per slice would be as high as possible to maximize the slope of each pixel's response. The time between irradiation and scanning was chosen to be shorter than the recommended 12 hs. The recommended 12‐hour gap between irradiation and scanning is, at least partly, to stabilize the irradiated gel during the time it takes to acquire a scan (MRI, CT,). It was desirable that the gel be a little unstable so that the CT dose could have its effect, which is the underlying principle of the zero‐scan method.

In this study, the numbers of scans have been reduced sharply compared to the previously reported case (from 360 down to 72).^(10)^ The reduction in the number of images is desirable for a high throughput if this method has to be employed in routine clinical dose verifications. Moreover, in some cases, heating of the CT scanner's X‐ray tube can be an issue and, thus, reduced acquisitions are advantageous. Also gel dosimeters which provide an increased CT signal^(12)^ would be advantageous for use with the zero‐scan method. The zero scan method has been shown to improve the image quality for the volumetric gel readout by use of multislice acquisitions.

## ACKNOWLEDGMENTS

Queensland Cancer Physics Collaborative is acknowledged for their support. The authors would like to acknowledge the help of Danielle Tyrell, Julie‐Anne Miller, and John Baines from Southern Area Radiation Oncology Services, Mater Hospital, Brisbane for gel irradiation. Paul Charles's contribution to this study was supported by the Australian Research Council, the Wesley Research Institute, Premion and the Queensland University of Technology, through linkage grant number LP110100401.
